# Supplementary material for: Bayesian distributional models of executive functioning
Source: Behav Res Methods. 2026 Oct 5;58(11):310. doi: 10.3758/s13428-026-03191-x (PMC13638740; doi:10.3758/s13428-026-03191-x)
Supplement: Supplementary file 1 [file 13428_2026_3191_MOESM1_ESM.pdf]

Bayesian Distributional Models of Executive Functioning

Robert Kasumba[1], Zeyu Lu[2], Dom CP Marticorena[3], Mingyang Zhong[3], Paul Beggs[3], Anja Pahor[4], Geetha Ramani[5], Imani Goffney[6], Susanne M Jaeggi[7], Aaron R Seitz[7], Jacob R Gardner[8], Dennis L Barbour[1,2,3]

[1]Division of Computing and Data Science, Washington University, 1 Brookings Drive, St. Louis, MO 63130
[2]Department of Computer Science and Engineering, Washington University, 1 Brookings Drive, St. Louis, MO 63130
[3]Department of Biomedical Engineering, Washington University, 1 Brookings Drive, St. Louis, MO 63130
[4]Department of Psychology, University of Maribor, 2000 Maribor, Slovenia
[5]Department of Human Development and Quantitative Methodology, University of Maryland, College Park, MD 20742-5025
[6]Department of Teaching and Learning, Policy and Leadership, University of Maryland, College Park, MD 20742-5025
[7]Department of Psychology, Northeastern University, Boston, MA 02115
[8]Department of Computer and Information Science, University of Pennsylvania, Philadelphia, PA 19104

Supplemental information for the manuscript with the same title

For Review Only

1. Supplementary analysis of model accuracy under matched observations

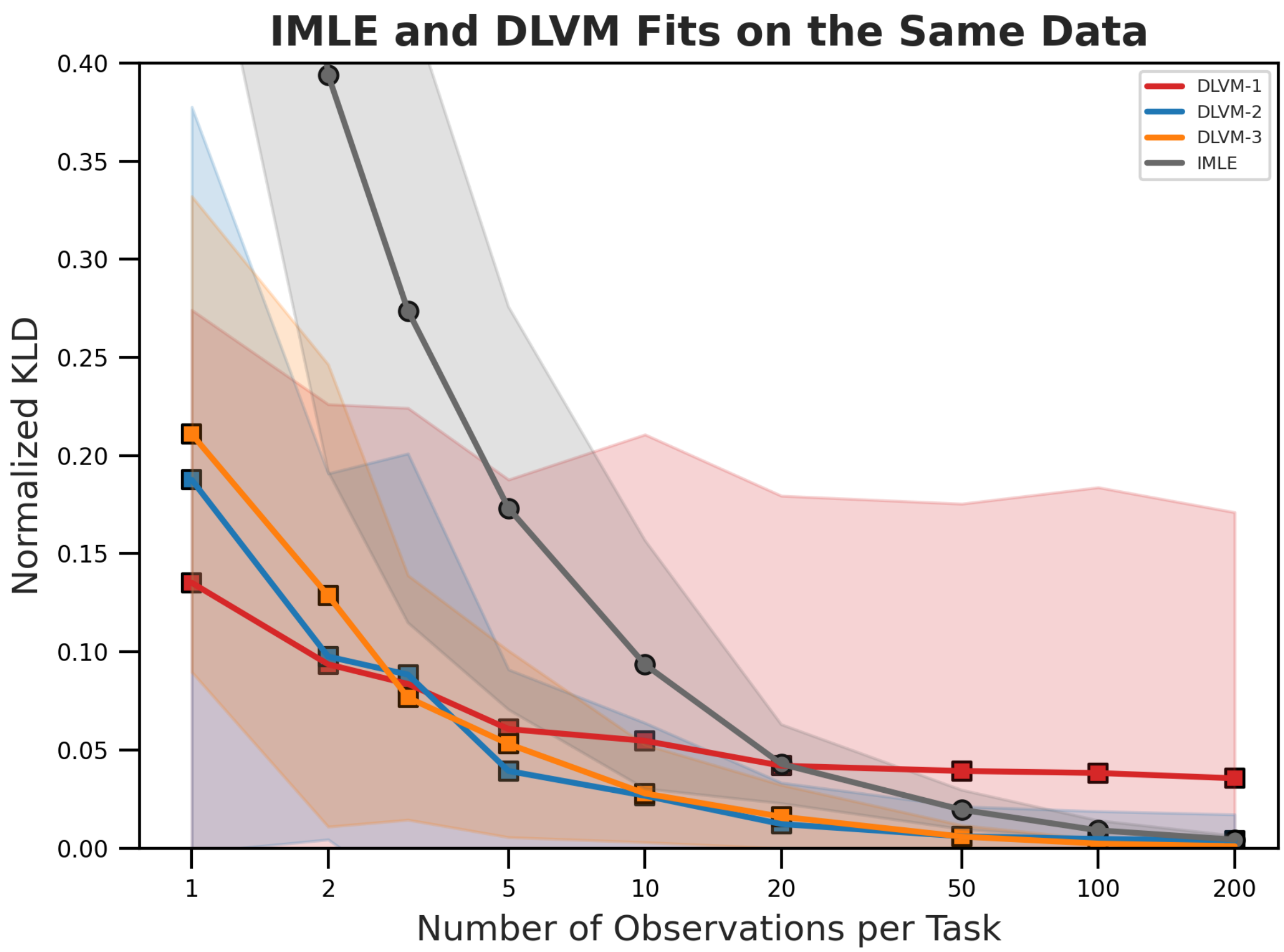

Figure S1: Mean ± standard deviation of DLVM (for three different model dimensionalities) and IMLE model accuracy (n = 88 each) as the number of observations per task increases.

2. Supplementary analysis of DALE trajectories after 240 observations

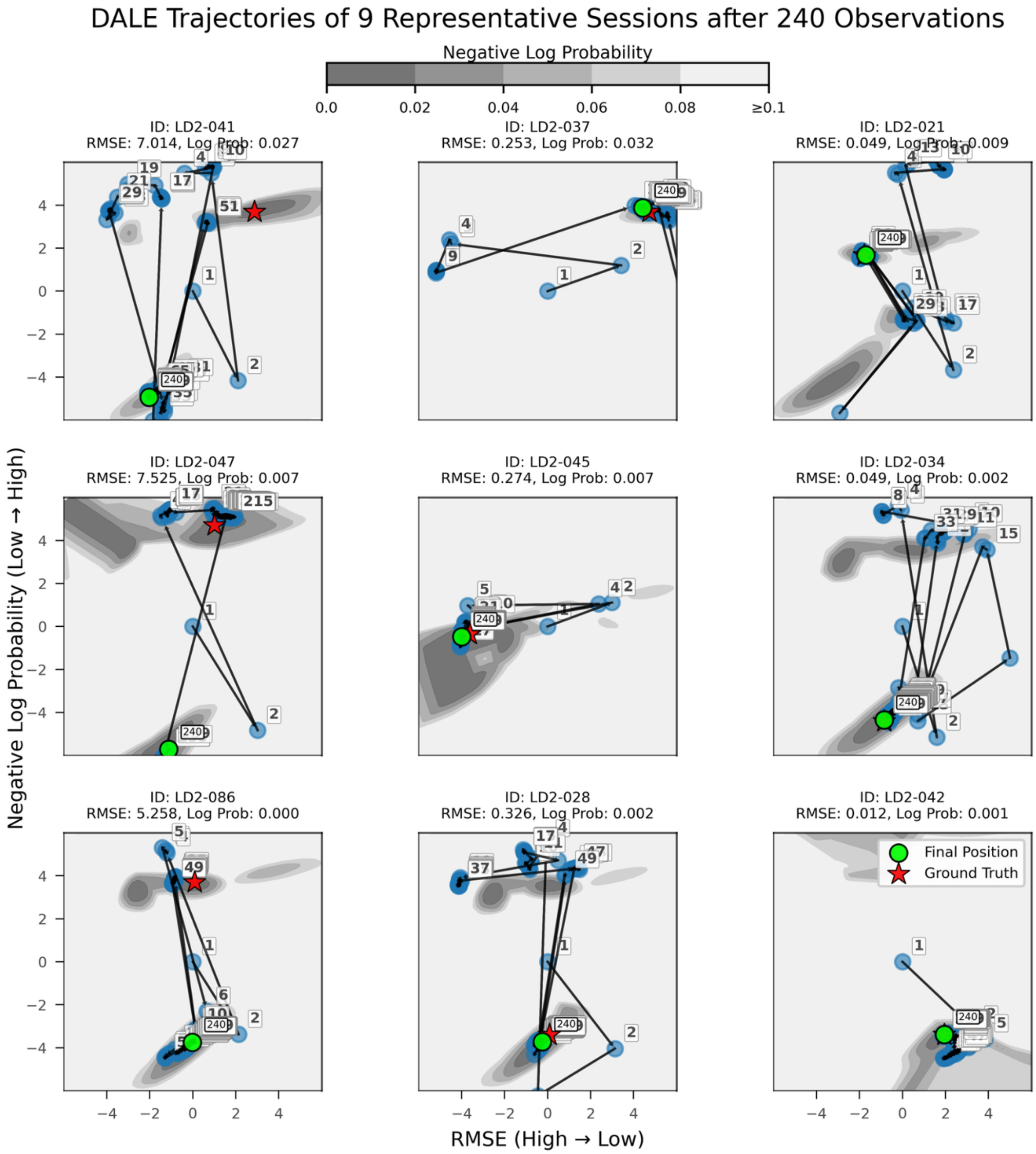

Figure S2: DLVM-2+DALE latent position updates as more data are collected for 3 representative simulated sessions based on the RMSE values between the ground truth position and the position estimated by DALE after accumulating 240 observations.

3. Supplementary analyses of latent dimensionality

3.1 Analysis of a One-Dimensional DLVM

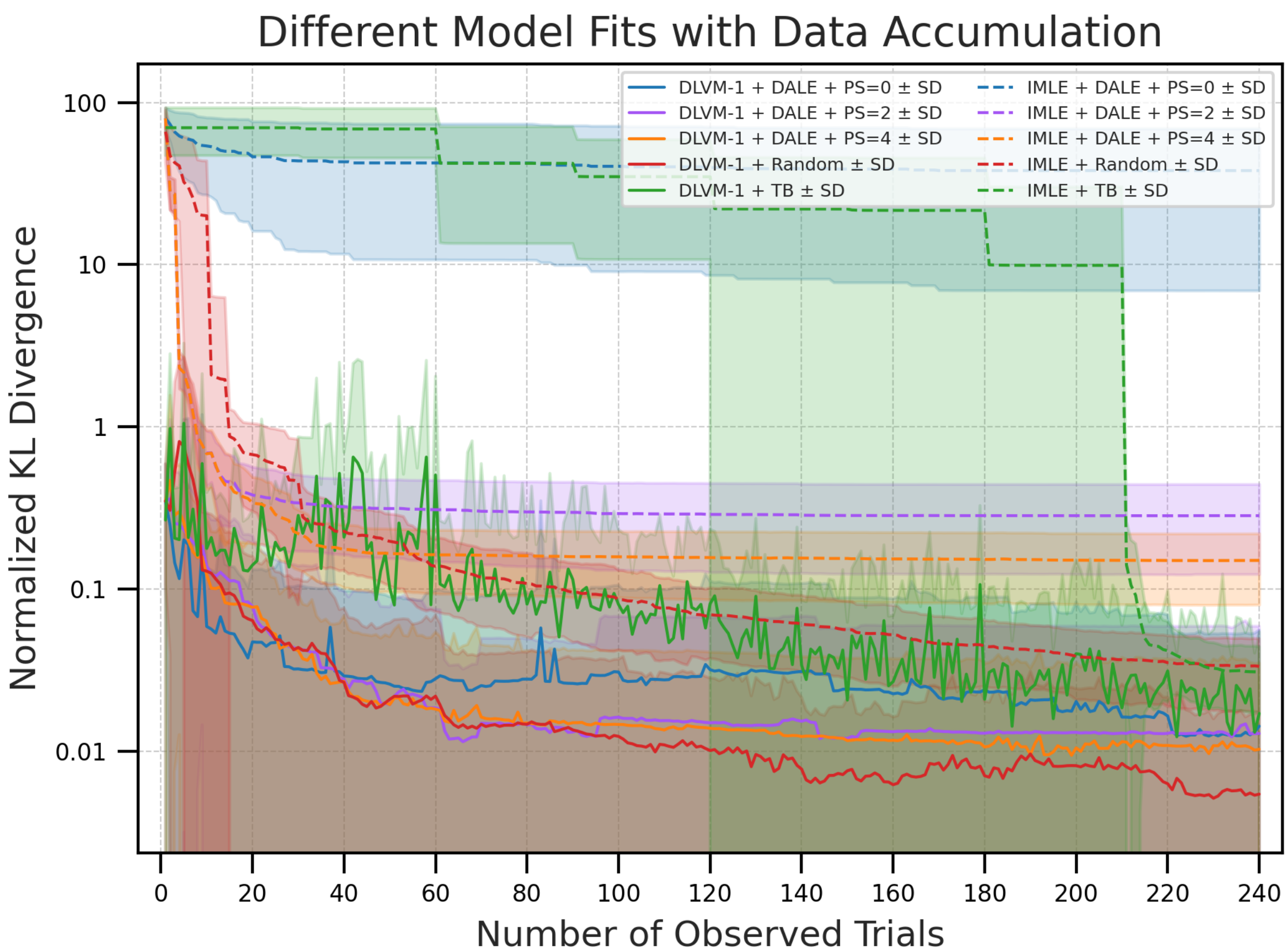

Figure S3: Performance of the different estimation methods and the sampling strategies for a 1D model. "PS=0" indicates that there was no primer sequence provided for DALE and thus the first sample was actively sampled, "PS=2" means that DALE was primed with 2 observations per task (16 total) and "PS=4", 4 observations per task (32 total) before active sampling started.

3.2 Analysis of a Two-Dimensional DLVM

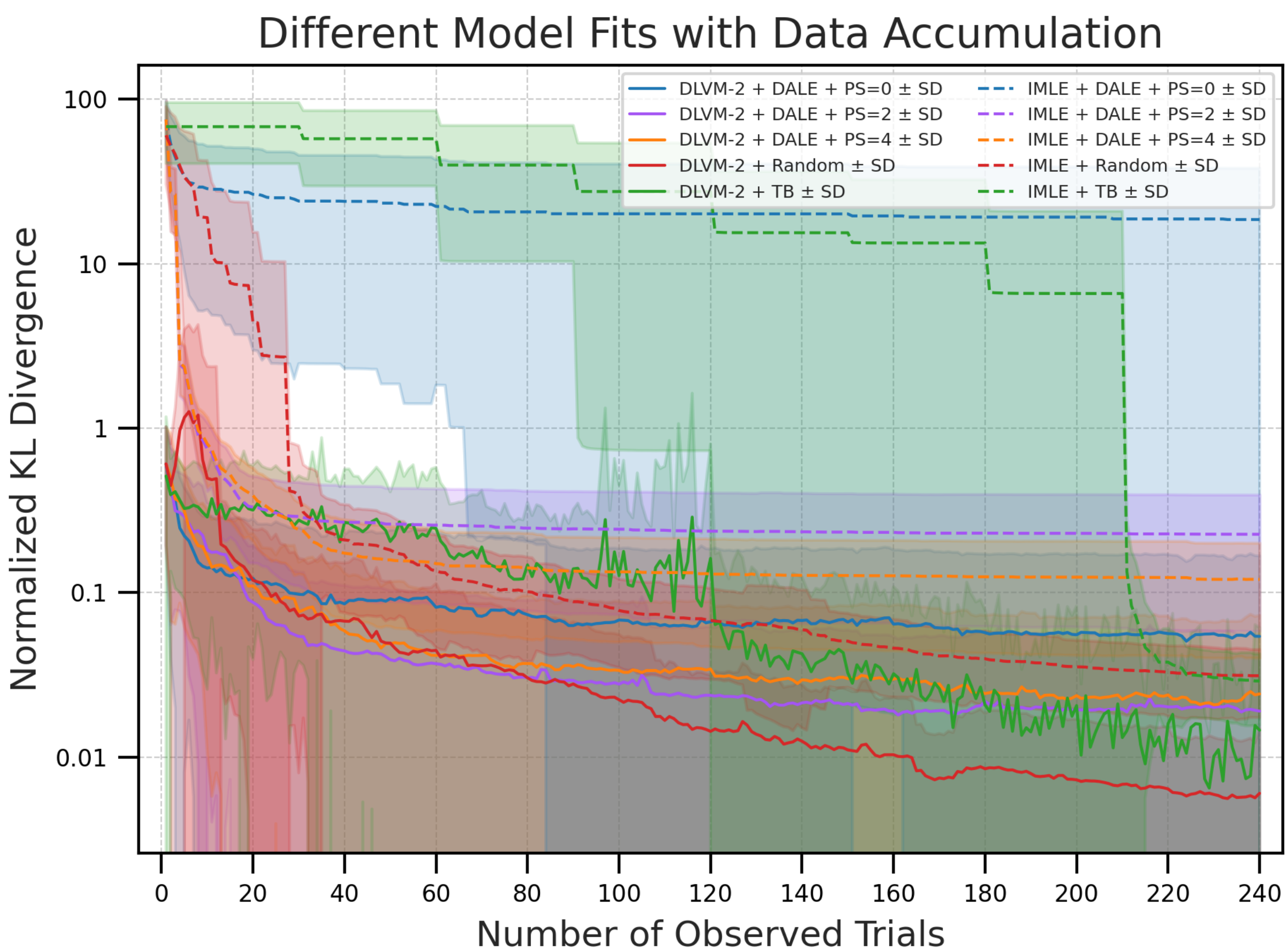

Figure S4: Performance of the different estimation methods and the sampling strategies for a 2D model. "PS=0" indicates that there was no primer sequence provided for DALE and thus the first sample was actively sampled, "PS=2" means that DALE was primed with 2 observations per task (16 total) and "PS=4", 4 observations per task (32 total) before active sampling started.

3.3 Analysis of a Three-Dimensional DLVM

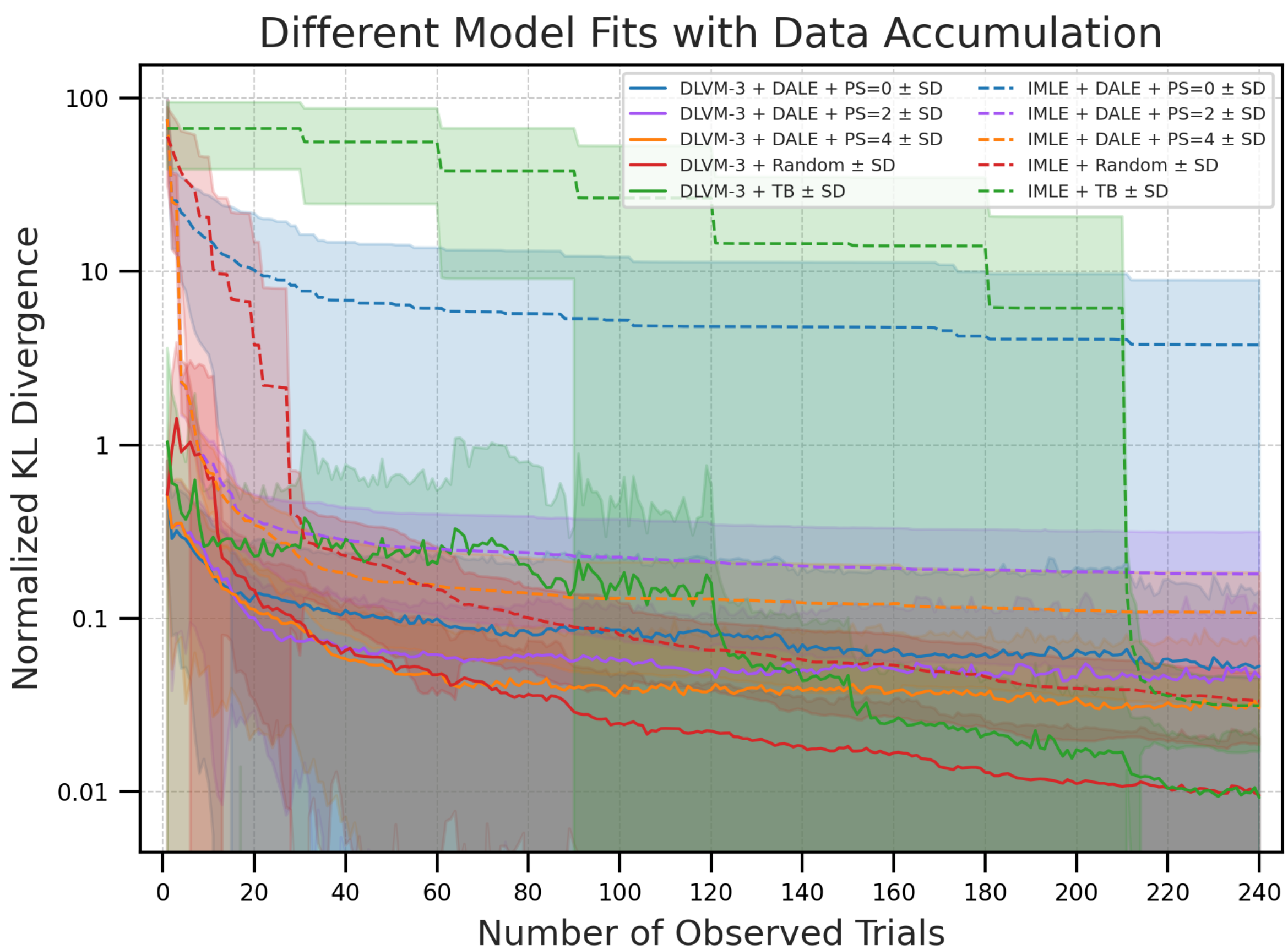

Figure S5: Performance of the different estimation methods and the sampling strategies for a 3D model. “PS=0” indicates that there was no primer sequence provided for DALE and thus the first sample was actively sampled, “PS=2” means that DALE was primed with 2 observations per task (16 total) and “PS=4”, 4 observations per task (32 total) before active sampling started.

4. Supplementary analyses using real data (IMLE fitted) distributions as the generative process

This analysis evaluates whether the primary results depend on using a DLVM-based generative process. Because the target distributions in this analysis are IMLE-fitted distributions, IMLE is expected to perform well when sufficient observations are available. The key question is whether DLVM and DALE retain advantages under sparse observations, which we observe.

4.1 Matched data observation performance for IMLE and DLVM

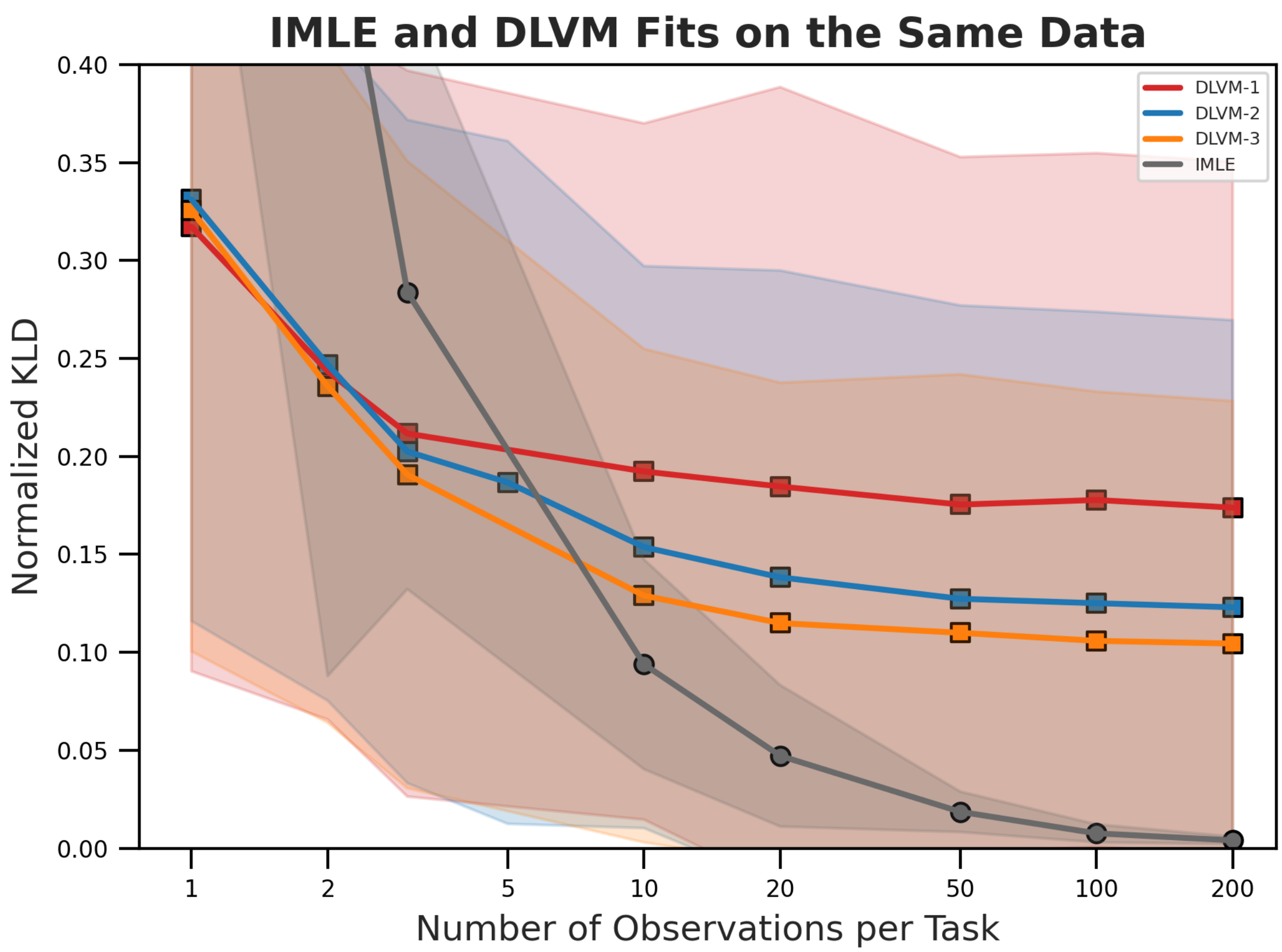

Only

Figure S6: Model Accuracy DLVM (for three different model dimensionalities) and IMLE when the observations are sampled from **IMLE-fitted distributions on real human data** (n = 88 each). The models aim to recover those IMLE-aligned distributional parameters.

4.2 One-dimensional DALE model

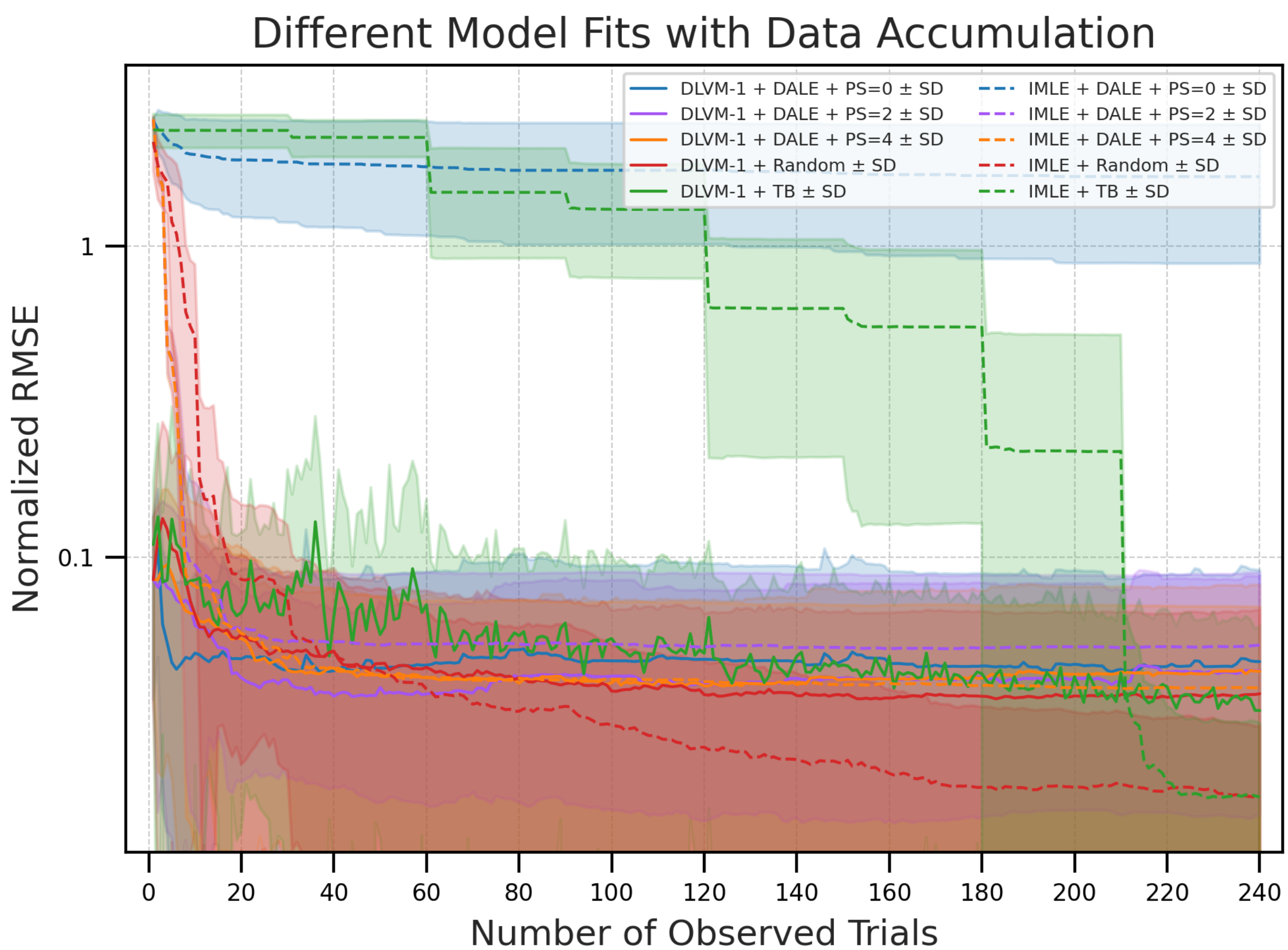

Figure S7: Performance of the different estimation methods and the sampling strategies for a 1D model when the observations are sampled from IMLE-fitted distributions on real human data.

4.3 Two-dimensional DALE model

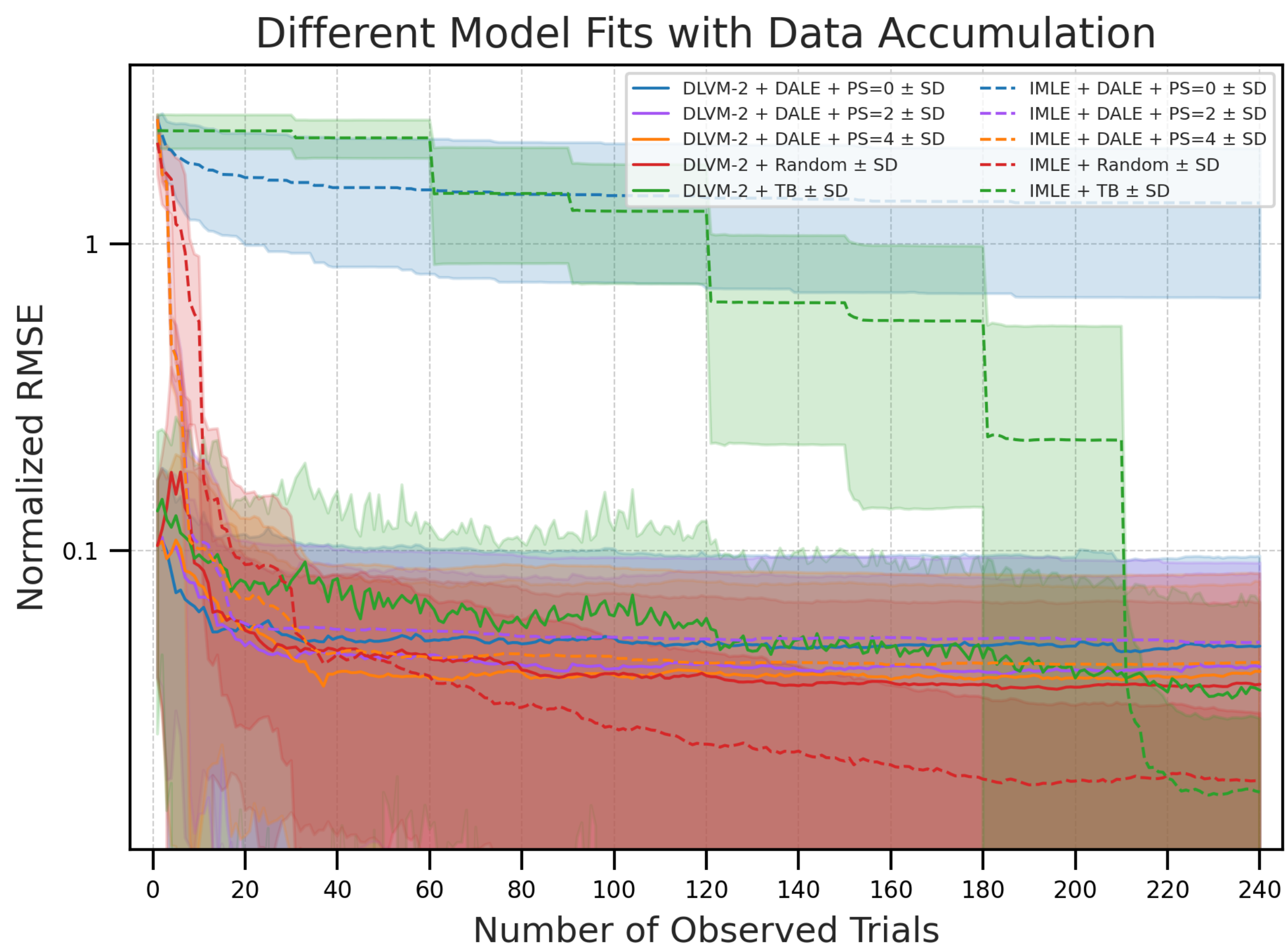

Figure S8: Performance of the different estimation methods and the sampling strategies for a 2D model when the observations are sampled from IMLE-fitted distributions on real human data.

4.4 Three-dimensional DALE model

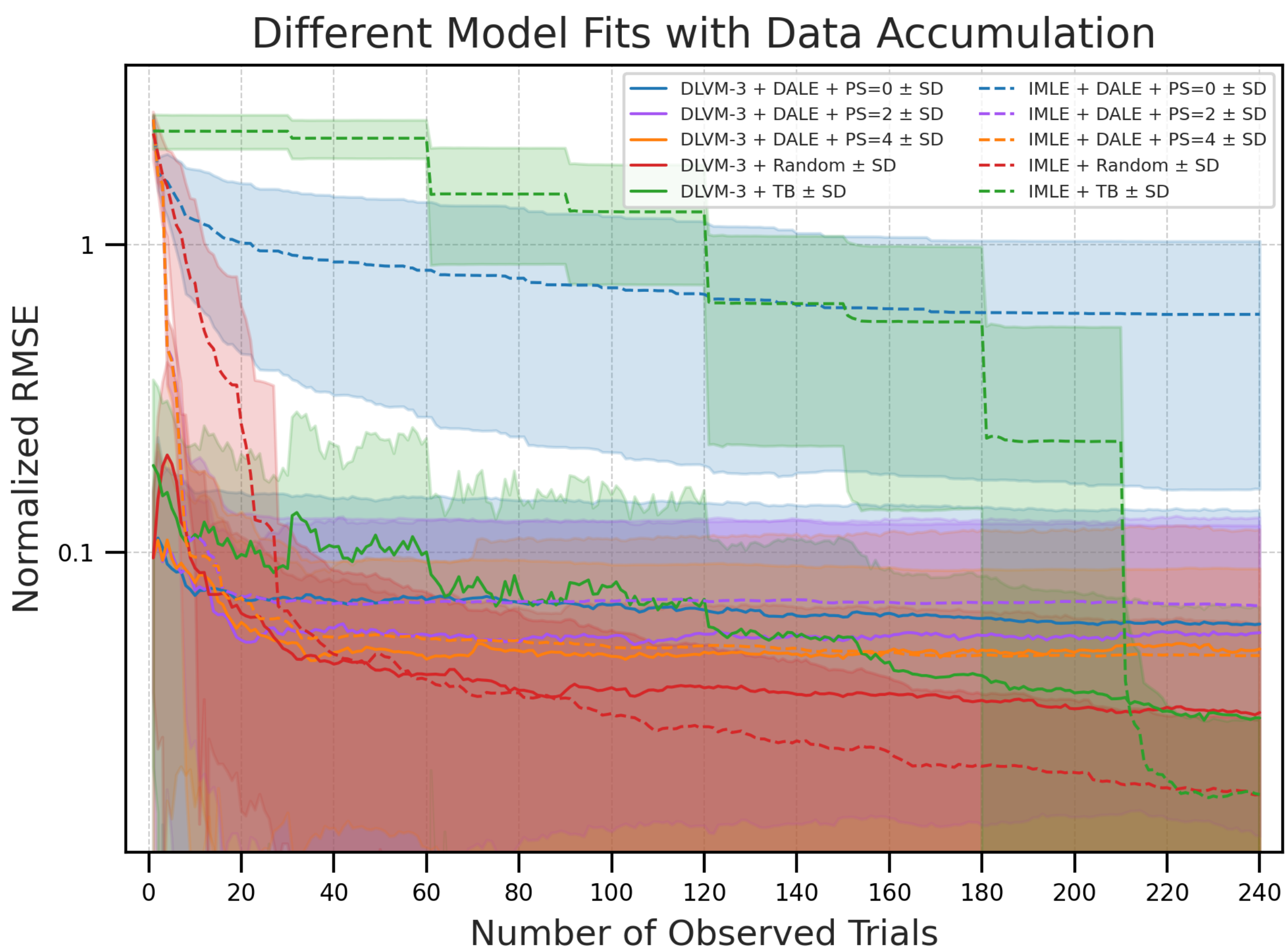

Figure S9: Performance of the different estimation methods and the sampling strategies for a 3D model when the observations are sampled from IMLE-fitted distributions on real human data.

5. Robustness analysis with outliers retained

The primary analysis used DLVM models trained with outlier sessions removed, retaining a total of n = 88 sessions. We retrained a 2-dimensional DLVM model without removing outlier sessions, a total of n = 101 sessions. Here we present this supplementary analysis. Figure S10 shows the learned latent space as well as the sampled synthetic individuals, and Figure S11 shows DALE performance. We evaluated DALE using 101 synthetic individuals using uniform sampling as in the primary analysis.

5.1 Learned DLVM latent space

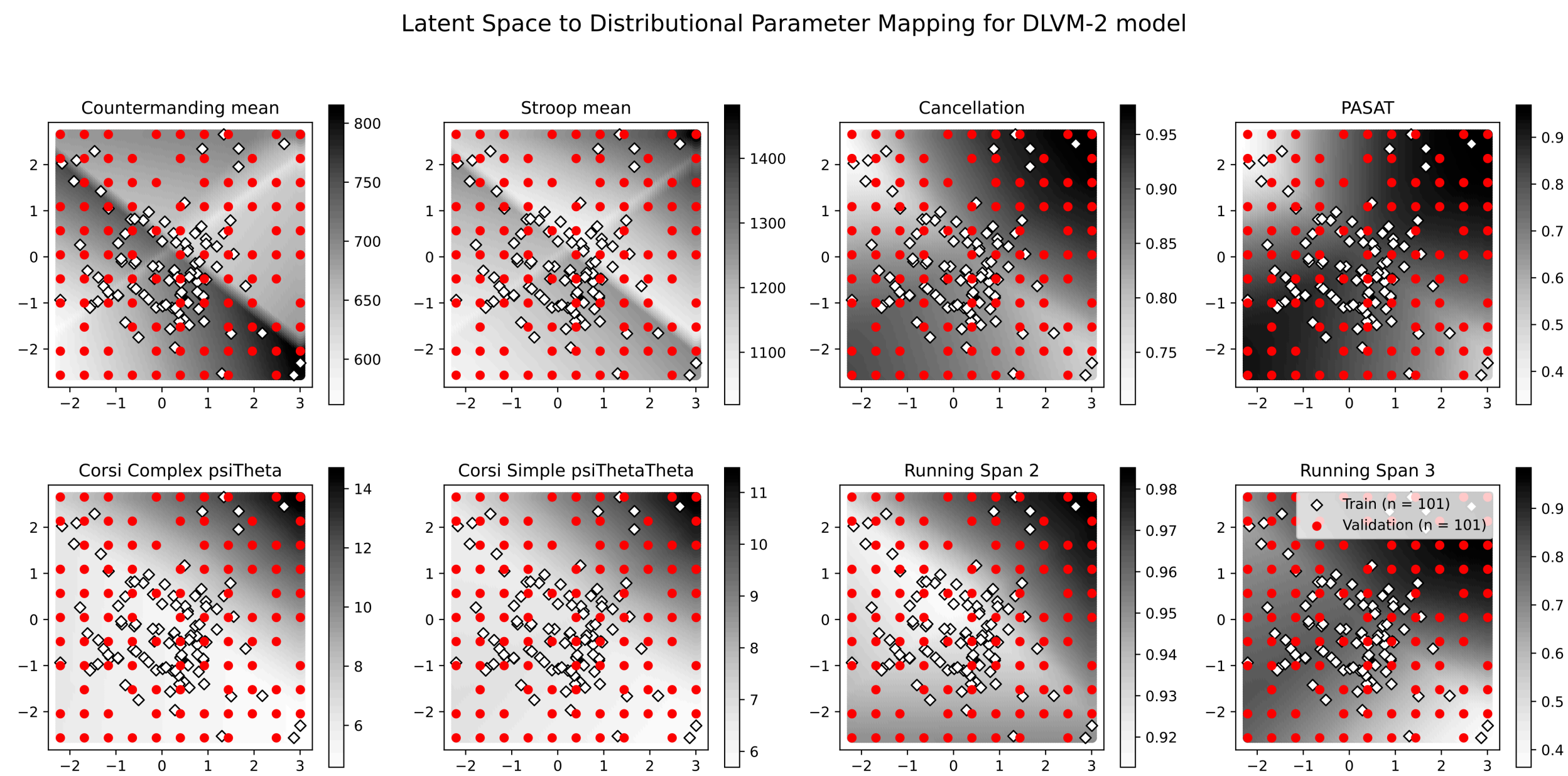

Figure S10: DLVM-2 latent-space-to-parameter mappings when outliers are retained. Compared with the primary DLVM-2 mapping shown in Figure 2, the expanded dataset produces visibly more locally irregular parameter surfaces while preserving the broad cross-task organization of the latent space. The additional sessions introduce sharper transitions and stronger nonlinear curvature in several mappings, most notably for Countermanding mean, Stroop mean, PASAT, and Running Span 3.

5.2 Analysis of Two-Dimensional DALE model

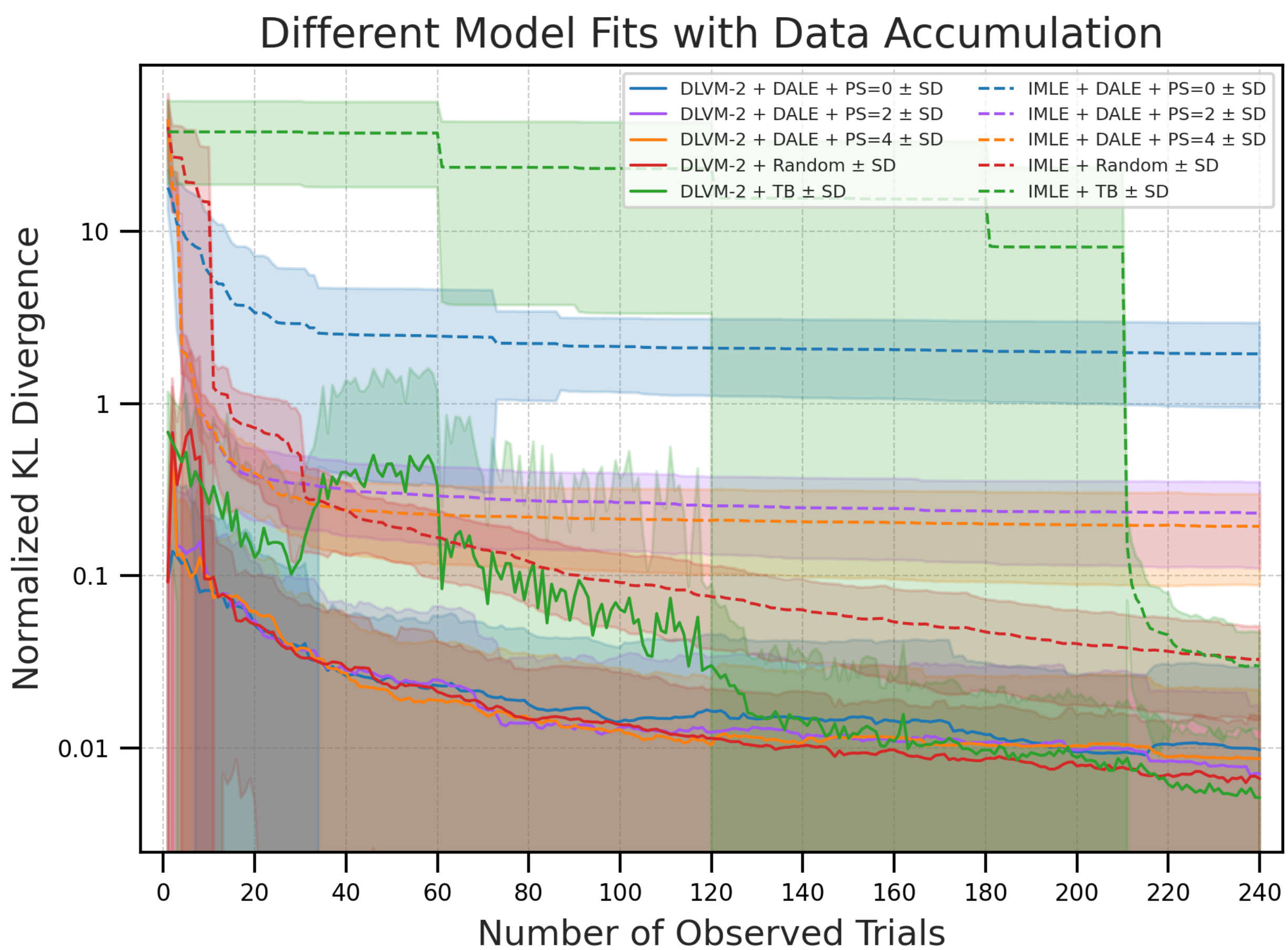

Figure S11: Performance of the different estimation methods and the sampling strategies for a 2D model when generative space was learned with outliers included. The general trend is consistent with the primary analysis, with DALE showing a slight advantage over random sampling within the first 100 observations. The advantage is less pronounced, however, compared to when the latent space is smoother.
